# Learning from failure feedback for subsequent task performance: A matter of personality?

**DOI:** 10.3389/fpsyg.2022.1032273

**Published:** 2022-12-15

**Authors:** Katharina Thiel, Thorsten Semrau

**Affiliations:** University of Trier, Trier, Germany

**Keywords:** failure learning, feedback, personality, conscientiousness, extraversion

## Abstract

The present study expands our knowledge of the differential consequences of failure feedback. Specifically, we conducted an online experiment to elaborate on how conscientiousness and extraversion contribute to explaining whether individuals learn from failure feedback for future task performance. In line with our hypotheses, we find that individuals who are highly conscientious and/or highly extraverted are more likely to learn from failure feedback than their counterparts. We discuss the implications of our study and derive practical implications.

## Introduction

“Success is not final; failure is not fatal: it is the courage to continue that counts.” – Winston Churchill.

Feedback is an integral part of the effective functioning of organizations (Cianci et al., 2010; Johnson and Connelly, 2014). Success feedback aims to encourage individuals to try even harder and set more challenging goals (Van-Dijk and Kluger, 2004; Cianci et al., 2010). Failure feedback–i.e., feedback indicating that one’s past performance does not meet expectations (Johnson and Connelly, 2014; Eskreis-Winkler and Fishbach, 2019)–is thought to create awareness for discrepancies between what has been achieved and what is expected and to motivate individuals to work harder, learn, and adapt their behavioral strategies (Van-Dijk and Kluger, 2004; Cianci et al., 2010).

However, previous research indicates that failure feedback often does not have the intended consequences (Kluger and DeNisi, 1996; Steelman and Rutkowski, 2004). In fact, a substantial number of studies reveal that failure feedback may have no effect at all and can even impair the subsequent performance of feedback recipients (Kluger and DeNisi, 1996; Cianci et al., 2010). This is because failure feedback not only provides guidance for learning and adaptation but can also evoke dissatisfaction (Belschak and Den Hartog, 2009), trigger defensive reactions and denial (Steelman and Rutkowski, 2004), and impede motivation and adaptation among feedback recipients (Yeager and Dweck, 2012).

Against this backdrop, research has begun to elaborate on what determines the consequences of failure feedback. Some studies reveal that contextual factors, such as the credibility and quality of the feedback provided (Steelman and Rutkowski, 2004), can help to explain how recipients react to failure feedback. Other studies have explored how recipients’ individual differences affect the consequences of feedback. Specifically, prior research has shown how differences in transient dispositions, such as regulatory focus (Van-Dijk and Kluger, 2004) and learning goal orientation (Dweck, 1986; Cianci et al., 2010), can affect whether recipients learn from failure feedback. The present study complements this prior research by elaborating on how conscientiousness and extraversion, two traits from the five-factor model (FFM) of personality (McCrae and Costa, 1987; McCrae and Costa, 2008), affect whether recipients learn from failure feedback for subsequent performance.

Focusing on conscientiousness and extraversion seems fruitful for several reasons. First, the two personality traits are widely recognized as particularly important in the work context (Barrick and Mount, 1991; Barrick et al., 2002). Second, both personality traits encompass aspects of achievement motivation and have been connected to the learning goal orientation of individuals (Payne et al., 2007; Wang and Erdheim, 2007), which has previously been identified as a relevant precursor to the consequences of failure feedback by previous research (Cianci et al., 2010).

When developing our hypotheses, we follow earlier research (Deichmann and Ende, 2013; Kc et al., 2013; Wilhelm et al., 2019) in adopting a learning perspective that focuses on the consequences of an observable learning input, i.e., failure feedback, for an observable outcome of the learning process, i.e., subsequent task performance. We test our hypotheses based on an online experiment with 47 individuals and find support for our theoretical ideas. With the insights generated, the present study advances our understanding of the differential consequences of failure feedback. Specifically, our study expands our knowledge of how the characteristics of the feedback recipient impact whether the potential for learning and improvement inherent in failure feedback is realized. Given the importance of feedback for goal setting and (re)directing efforts and that failure is commonplace in organizational contexts (Morgenroth and Schaller, 2010; Dahlin et al., 2018), our study also has practical implications.

## Theory and hypotheses

### Learning from failure feedback

Failure is an outcome that falls short of what is expected or desired (Rasmussen, 1982; Reason, 1990; Sitkin, 1992; Zhao and Olivera, 2006), and failure feedback is feedback indicating to recipients that their performance did not meet expectations (Johnson and Connelly, 2014; Eskreis-Winkler and Fishbach, 2019).

Failure feedback is widely recognized as crucial for securing long-term effectiveness in organizational contexts, as it can help redirect the efforts of individuals and motivate them to learn and improve their performance (Johnson and Connelly, 2014). However, failure feedback does not always have these intended consequences (Kluger and DeNisi, 1996). Clearly, failure feedback can be of developmental value, as it may help the recipient understand expectations and indicate the potential causes for failure, which can guide adaptations to behavioral strategies (Van-Dijk and Kluger, 2004; Cianci et al., 2010). However, failure feedback is also always unpleasant (Steelman and Rutkowski, 2004), creates dissatisfaction (Belschak and Den Hartog, 2009) and can be perceived as a threat to one’s self-esteem (Cianci et al., 2010). As such, failure feedback can evoke defensive reactions (Cochran and Tesser, 1996; Soman and Cheema, 2004; Belschak and Den Hartog, 2009), and recipients may infer that they are perceived as not committed to the task at hand or that they lack aptitude (Fishbach and Finkelstein, 2012), which can compromise their motivation to learn and discourage adaptation and improvement (Yeager and Dweck, 2012).

Mirroring this ambiguity, studies reveal that failure feedback can help to stimulate learning and improvement but may also impair or have no effect on subsequence performance (Kluger and DeNisi, 1996; Steelman and Rutkowski, 2004). Scholars have thus called for further research to identify what determines the performance implications of failure feedback (Steelman and Rutkowski, 2004; Cianci et al., 2010). Responding to these scholarly calls, we elaborate on why we expect the personality of feedback recipients to play an important role in this regard.

### Personality and learning from failure feedback

Personality refers to the relatively stable differences in the way individuals feel, think and behave (Digman, 1990; McCrae and Costa, 1997; Tasselli et al., 2018). Drawing on the five-factor model (FFM) of personality (McCrae and Costa, 1987; McCrae and Costa, 2008) as an organizing framework, previous research has firmly established that personality affects individual action and outcomes relevant to work contexts (Barrick and Mount, 1991; Tett et al., 1991).

**Figure 1:**
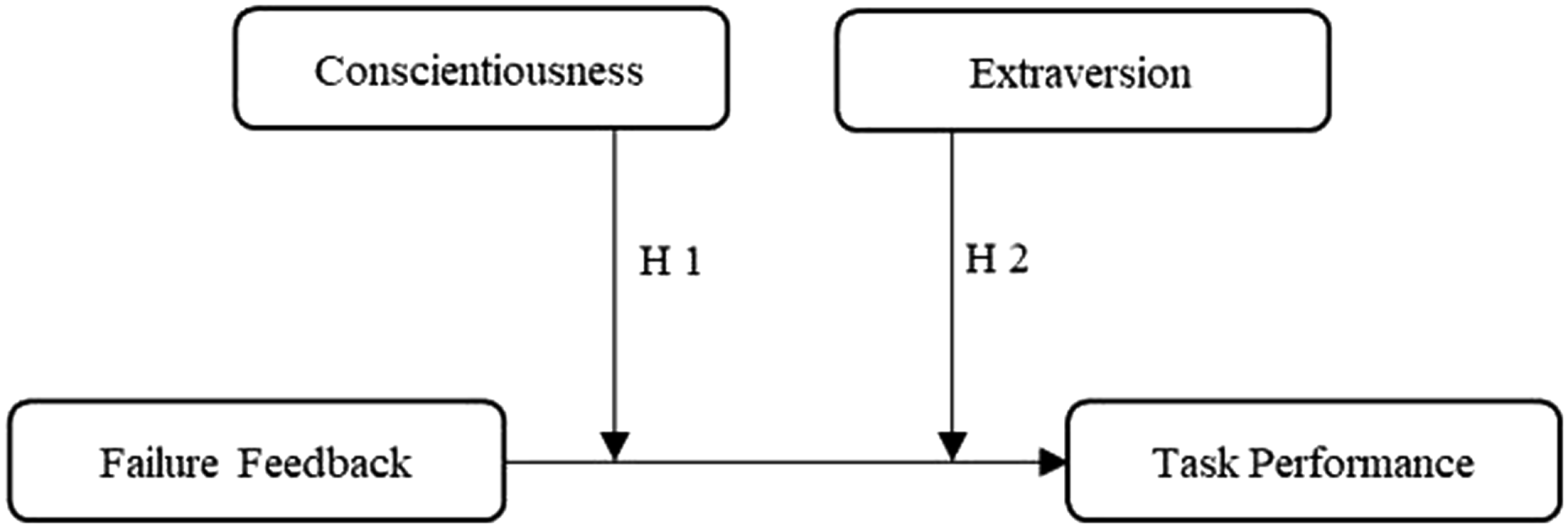
Conceptual model.

Building on insights generated by previous research, we subsequently develop arguments to suggest that two of the FFM personality traits–i.e., conscientiousness and extraversion–shape whether individuals learn from failure feedback. In doing so, we follow previous research (Deichmann and Ende, 2013; Kc et al., 2013; Wilhelm et al., 2019) and adopt a learning perspective that focuses on the consequences of failure feedback–an observable learning input–for subsequent task performance–an observable learning outcome (see Figure 1).

### Conscientiousness and failure feedback learning

Conscientiousness reflects the extent to which individuals are ambitious, hard-working, perseverant, and disciplined in focusing on goals (McCrae and Costa, 1987; Barrick and Mount, 1993; Rothmann and Coetzer, 2003) As such, conscientiousness is widely recognized as the most consistent predictor for performance in various work contexts (Barrick and Mount, 1991; Barrick et al., 2002) and has been connected to individual differences in learning goal orientation by previous research (Payne et al., 2007; Wang and Erdheim, 2007). Building on these insights, we anticipate that conscientiousness plays a significant role in explaining whether individuals learn from failure feedback. Specifically, we suggest that conscientiousness will stimulate learning, as reflected in the relationship between failure feedback and subsequent task performance.

When conscientiousness is high, individuals have a strong desire to achieve, maintain high performance standards, and tend to strive for perfection (Barrick et al., 2002; Curşeu et al., 2019). Confronted with feedback indicating that an outcome generated does not meet expectations, highly conscientious individuals will thus likely feel tension and the pressure to improve (Cianci et al., 2010) and take steps to learn and hone their capabilities (Smither et al., 2005). Given that highly conscientious individuals tend to be well organized (Roberts et al., 2009), they should also be able to effectively orchestrate their learning activities to enhance their future performance.

In contrast, low-conscientious individuals confronted with failure feedback are less likely to feel inclined to learn and improve. When conscientiousness is low, individuals are neither particularly committed nor persistent in goal achievement (Colbert et al., 2004). Compared to their high-conscientious counterparts, they are thus less likely to feel that they have to meet performance expectations and maintain high levels of effort (Cianci et al., 2010), which compromises their motivation to learn from failure feedback. Moreover, the working style of low-conscientiousness individuals (Roberts et al., 2009) may prevent them from effectively analyzing the causes of failure and reorganizing their behavior strategies to effectively learn and improve their future performance.

Based on these lines of reasoning, we submit that conscientiousness affects whether individuals learn from failure feedback for subsequent performance. Therefore, we propose the following:

*Hypothesis 1*: Conscientiousness positively moderates the link between failure feedback and subsequent task performance.

### Extraversion and failure feedback learning

Like conscientiousness, we also expect extraversion to affect whether individuals learn from failure feedback for subsequent task performance. Extraverts are social and assertive, seek external stimuli, and desire to excel above others (Barrick and Mount, 1991; Depue and Collins, 1999; Ashton et al., 2002). As such, previous research has found extraversion to predict success in various job roles (Barrick and Mount, 1991) and to relate to the learning goal orientation of individuals (Wang and Erdheim, 2007). Building on these insights, we argue that extraversion positively moderates the link between failure feedback and subsequent task performance.

Extraverts tend to be self-confident, action-oriented, motivated by competition and opportunities to demonstrate competence (Barrick and Mount, 1991; Barrick et al., 2002), and seek out and enjoy change (Bono and Judge, 2004). As such, extraverts are unlikely to feel discouraged when confronted with failure feedback. Instead, their self-confidence and desire to be perceived as competent fuel their motivation to learn and adapt to realize superior performance in the future.

In contrast, introverts, i.e., individuals scoring low on extraversion, likely lack what it takes to constructively deal with feedback indicating that their performance does not meet expectations. Compared to their extraverted counterparts, introverts tend to be less self-confident and action-oriented (Barrick and Mount, 1991) and do not have a strong desire to outperform others (Barrick et al., 2002). When confronted with failure feedback, introverts are thus less likely to feel the urge to adapt and improve and are more likely to feel discouraged, which is counterproductive for learning and improvement.

In line with these arguments, we thus expect that extraverts are more likely than their introverted counterparts to learn from failure feedback for subsequent task performance. Therefore, we hypothesize the following:

*Hypothesis 2*: Extraversion positively moderates the relationship between failure feedback and subsequent task performance.

## Materials and methods

### Participants and procedure

To test our theorizing, we designed an online experiment comprising two phases. In the first phase, the participants completed a survey and answered questions about their personality and their performance expectations related to the task presented and our other control variables. Then, the participants had 5 min to work on the number series task from the A-form of the German Intelligence-Structure-Test 2000-R (IST 2000-R) (Liepmann et al., 2007). The number series task presents 20 series of numbers formed according to a specific rule, which must be completed by writing down the next number. Afterward, the participants received false feedback on how they performed. Specifically, the participants were randomly assigned to one of two feedback conditions: failure feedback (“your result is below expectations”), which was coded 1, or success feedback (“your result is above expectations”), which was coded 0. All participants were then asked to answer a question serving as a manipulation check. A few hours later, the participants received an e-mail with instructions on how they could learn to best approach number sequence tasks if they wanted to. Seven days later, the study participants who completed phase one of our experiment were invited to participate in phase two. In this phase, the participants were provided 5 min to solve 20 number sequences from the C-form of the IST 2000-R (Liepmann et al., 2007). Afterward, the participants were debriefed and thanked for their participation.

The participants in our online experiment were invited *via* the daily student newsletter at Trier University. Participation was voluntary and anonymous. Participants were naïve to the purpose of the study and provided written informed consent.

In total, 104 individuals participated in the first phase of our experiment. Of these participants, 57 (59%) also participated in phase two, resulting in a dropout rate that is comparable to other studies with a 1-week time lag between study phases (Dormann and Griffin, 2015). An additional 10 participants were eliminated because of unmatched participation codes, leaving us with data from 47 participants for our hypothesis testing. On average, the participants were 23.5 years old (ranging from 19 to 35 years), and 34 were female.

### Measures

#### Task performance

To capture *task performance* following failure feedback, we utilized the number of participants’ correct responses to the number series task of the C-form of the IST 2000-R (Liepmann et al., 2007) in the second phase of our experiment. The mean score was 10.19 (SD = 4.06).

#### Conscientiousness

*Conscientiousness* was measured using the six items from the German version of the BFI-2-S (Soto and John, 2017). A sample item is “I see myself as someone who is persistent, works until the task is finished.” The participants responded on a 5-point Likert scale ranging from 1 (strongly disagree) to 5 (strongly agree). Given the high level of internal consistency observed (Cronbach’s alpha = 0.80), the item scores were aggregated into scale scores.

#### Extraversion

Similar to conscientiousness, we also measured *extraversion* using the six items of the German version of the BFI-2-S (Soto and John, 2017). A sample item is “I see myself as someone who is outgoing, sociable.” The respondents answered each item using a Likert scale ranging from 1 (strongly disagree) to 5 (strongly agree). We observed a high level of internal consistency among the six items (Cronbach’s alpha of 0.77) and thus aggregated them into scale scores.

#### Manipulation check

We assessed the effect of our failure feedback manipulation by asking participants to indicate their *satisfaction* with their own performance in solving number sequences. Response options ranged from 1 (very dissatisfied) to 5 (very satisfied).

#### Controls

We incorporated several controls into our analyses. First, we accounted for differences in *performance expectations* related to the task at hand. To do so, we asked the participants to assess their aptitude in solving number sequences based on a 5-point Likert scale with responses ranging from 1 (highly below average) to 5 (highly above average). Given that gender may affect how individuals react to success and failure experiences (Beyer, 1998; Simon and Nath, 2004), we also controlled for the participants’ *gender* (0 = male, 1 = female). Moreover, we controlled for the participants’ *age* (in years) and further included a variable indicating whether the participants had obtained a *bachelor’s* degree (0 = no, 1 = yes). We additionally accounted for individual differences related to the FFM variables that were not subject to our theorizing, i.e., *agreeableness*, *openness to experience* and *neuroticism*. Based on the German version of the BFI-2-S (Soto and John, 2017), we captured each of these personality traits with six items. For all items, the participants indicated their responses on a Likert scale ranging from 1 (strongly disagree) to 5 (strongly agree). Sample items are “I see myself as someone who assumes the best of people” (agreeableness), “I see myself as someone who is original, comes up with new ideas” (openness to experience) and “I see myself as someone who worries a lot” (neuroticism). For every six-item set, we observed a high level of consistency (Cronbach’s *α* = 0.70 for agreeableness, 0.80 for openness to experience and 0.77 for neuroticism) and thus combined the items to scale values.

### Analyses and results

An examination of responses to our manipulation check revealed that, on average, the study participants receiving failure feedback reported lower levels of satisfaction (*M* = 2.25, SD = 0.98) than those receiving success feedback (*M* = 3.91, SD = 0.66). The results from an independent samples *t*-test confirmed that the two means differed significantly (*t*(45) = −6.72, *p* = 0.000).

Table 1 presents the means, standard deviations, and correlations of the studied variables.

**Table 1:** Means, standard deviations, and correlations.

Variable		Mean	SD	1	2	3	4	5	6	7	8	9	10
1	Task performance	10.191	4.068										
2	Age	23.52	2.890	−0.122									
3	Gender^a^	0.723	0.452	0.159	−0.216								
4	Bachelor	0.383	0.491	0.278^†^	0.279^†^	−0.100							
5	Performance expectations	3.234	0.728	0.425**	−0.125	−0.129	0.169						
6	Conscientiousness	3.645	0.603	0.047	−0.122	0.044	0.114	−0.112					
7	Extraversion	3.358	0.671	0.031	0.131	−0.466**	0.212	0.218	0.338*				
8	Agreeableness	3.691	0.646	−0.049	0.007	0.111	0.083	0.149	0.320*	0.189			
9	Neuroticism	2.822	0.676	−0.144	−0.006	0.239	−0.205	−0.362*	−0.100	−0.374**	−0.241		
10	Openness	3.524	0.618	−0.225	−0.147	0.025	−0.163	−0.182	−0.079	−0.116	−0.035	−0.022	
11	Failure feedback	0.510	0.505	0.025	−0.117	−0.034	−0.017	−0.095	−0.130^†^	−0.263	−0.195	0.239	−0.123

*N* = 47; SD = standard deviation.

^†^*p* < 0.10; *p < 0.05; **p < 0.01.

a Dummy coded: 1 = female, 0 = male.

Table 2 shows the results from our regression analyses conducted with SPSS 27. To facilitate the interpretation of coefficients, we standardized all our nonbinary predictors before entering them into our regression models.

**Table 2:** Results from analyses.

	Task performance							
	Model 1		Model 2		Model 3		Model 4	
	Coefficient	SE	Coefficient	SE	Coefficient	SE	Coefficient	SE
Intercept	7.666**	1.448	7.365**	1.356	7.846**	1.354	7.590**	1.323
Age	−0.784	0.507	−0.969^†^	0.488	−0.797	0.481	−0.940^†^	0.474
Gender	2.486^†^	1.434	2.925*	1.374	2.467^†^	1.360	2.817*	1.337
Bachelor	1.177	1.194	1.228	1.133	1.402	1.138	1.394	1.105
Performance expectations	1.649*	0.612	2.091*	0.663	2.087*	0.663	2.221*	0.649
Conscientiousness	0.638	0.632	−0.532	0.831	0.844	0.638	−0.144	0.838
Extraversion	0.077	0.732	−0.053	0.665	−1.611	1.003	−1.352	0.986
Agreeableness	−0.752	0.600	−1.034^†^	0.541	−0.833	0.522	−1.077*	0.526
Neuroticism	−0.430	0.643	−0.911	0.669	−0.524	0.634	−0.875	0.648
Openness	−0.659	0.579	−0.794	0.618	−0.589	0.622	−0.663	0.606
Failure feedback	0.612	1.160	0.879	1.106	0.643	1.101	0.848	1.077
Conscientiousness * Failure feedback			2.745*	1.224			2.048^†^	1.168
Extraversion * Failure feedback					2.438*	1.091	2.011^†^	1.153

*N* = 47; SE = standard error.

^†^*p* < 0.10; *p < 0.05; **p < 0.01.

Model 1 includes our control variables and tests for a potential uniform effect of failure feedback on subsequent performance. In line with prior research (Kluger and DeNisi, 1996; Steelman and Rutkowski, 2004), we found no uniform effect of *failure feedback* (*β* = 0.612, *p* = 0.601, Model 1) to indicate that our study participants generally learned from failure feedback for subsequent task performance.

To test our hypotheses, we first performed simple moderation analyses using the SPSS PROCESS macro (Hayes, 2018) and calculated coefficients and standard errors. The results of these analyses are shown in Table 2, Models 2 and 3.

**Figure 2:**
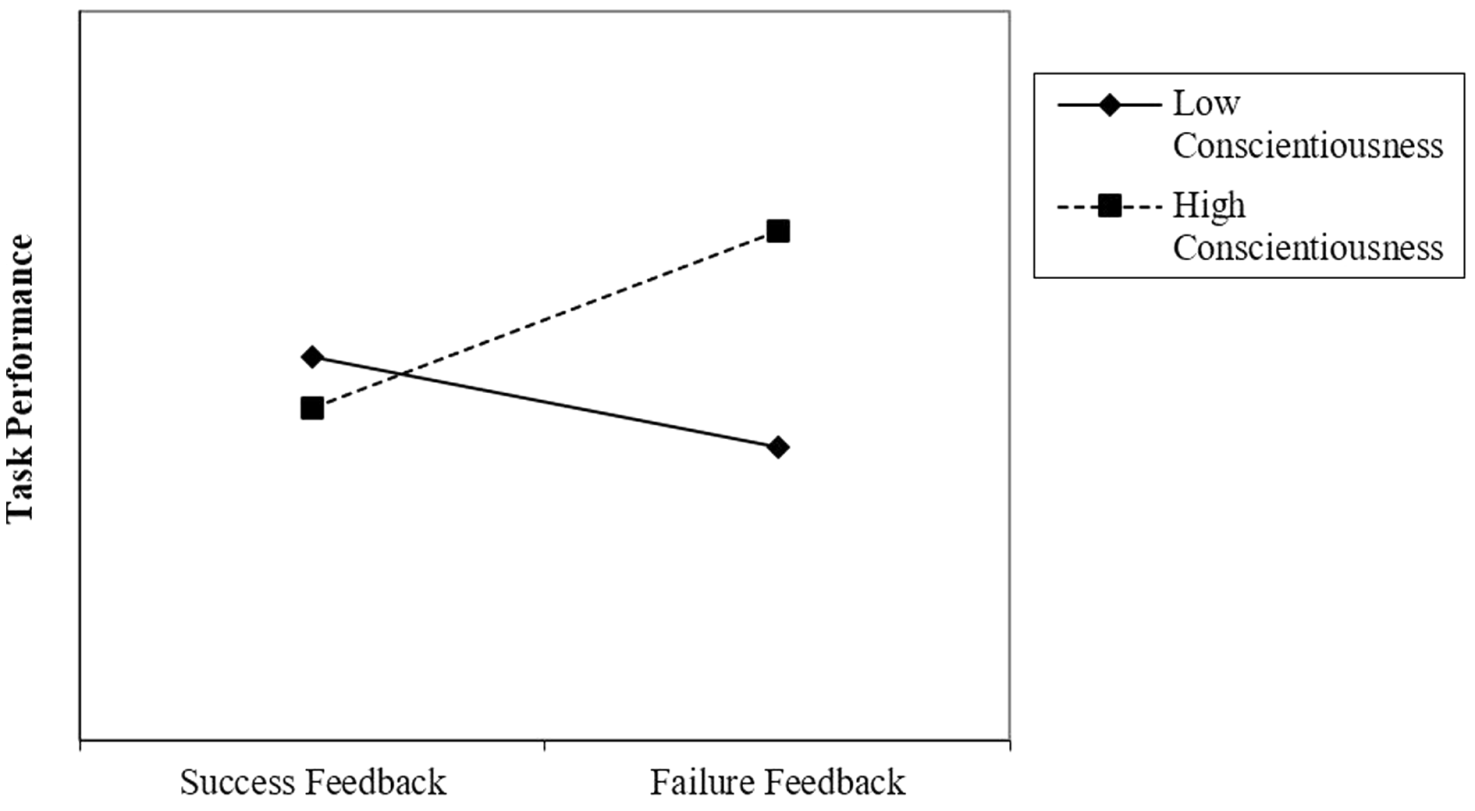
The moderating effect of conscientiousness.

Hypothesis 1 suggested that conscientiousness has a positive moderating effect on the relationship between failure feedback and subsequent task performance. Providing evidence in support of Hypothesis 1, our analyses reveal a positive interaction effect between failure feedback and conscientiousness (*β* = 2.745, SE = 1.224, 95% CI = [0.260, 5.230], *t* = 2.24, *p* = 0.031). The conditional effects of failure feedback on subsequent task performance at one standard deviation above (high) and below (low) the mean level of conscientiousness shown in Table 3 facilitate the interpretation of this result. In line with our theoretical arguments, we find a positive effect of failure feedback on subsequent task performance when conscientiousness is high (*b*_high_ = 3.324; *p* = 0.033), while the effect of failure feedback is nonsignificant and negative when conscientiousness is low (*b*_low_ = −1.865; *p* = 0.235). Figure 2 illustrates these results.

**Table 3:** Results from conditional effects analyses.

			Task performance
			Simple slope
			Failure feedback
Conscientiousness	High	+1 SD	3.324*
	Low	−1 SD	−1.865
Extraversion	High	+1 SD	3.081^†^
	Low	−1 SD	−1.795

^†^*p* < 0.10; *p < 0.05; **p < 0.01.

**Figure 3:**
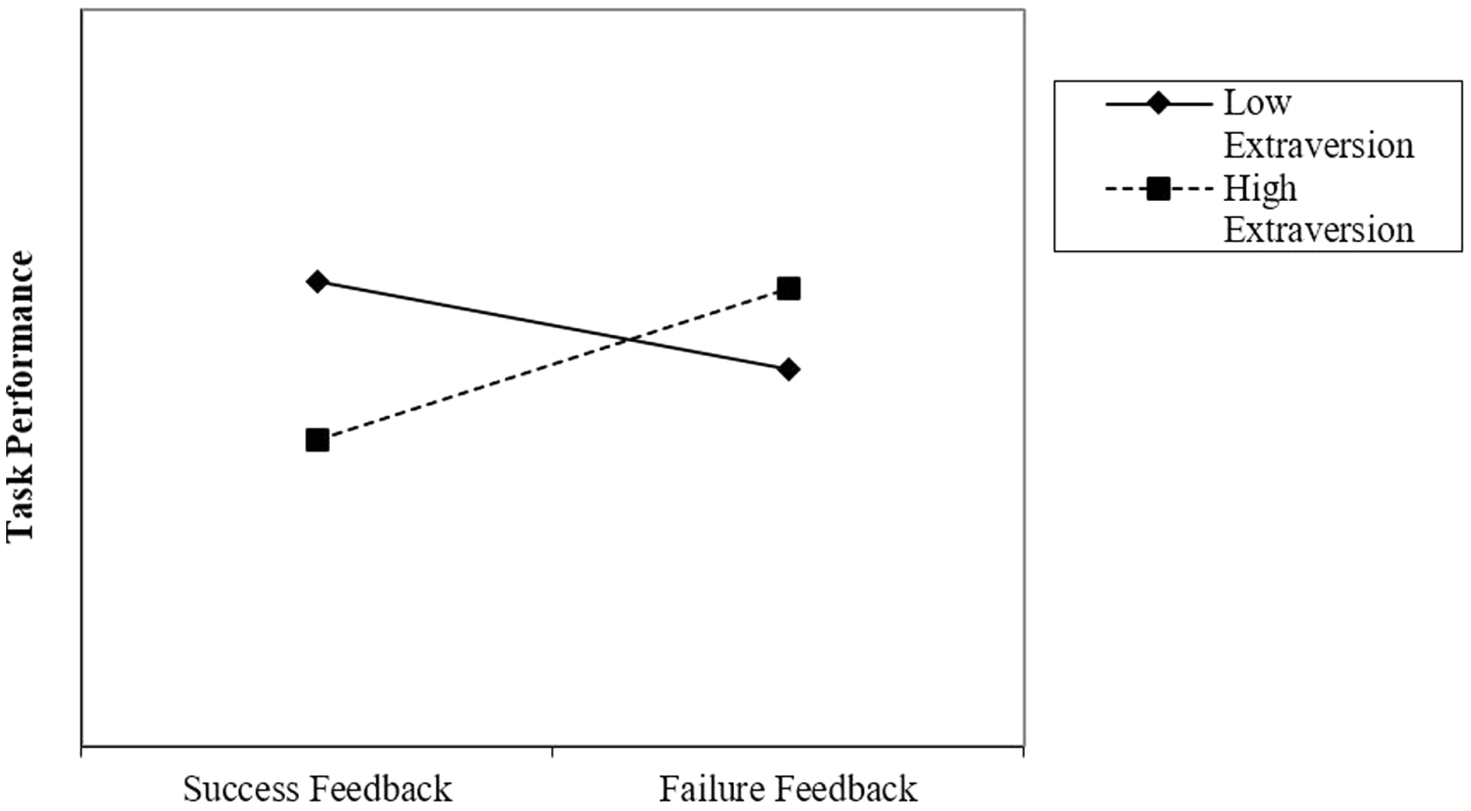
The moderating effect of extraversion.

Hypothesis 2 suggested that extraversion has a positive moderating effect on the relationship between failure feedback and subsequent task performance. In support of Hypothesis 2, our analyses reveal a positive interaction effect between failure feedback and extraversion (*β* = 2.438, SE = 1.091, 95% CI = [0.224, 4.623], *t* = 2.24, *p* = 0.031). The conditional effects of failure feedback on subsequent task performance at one standard deviation above (high) and below (low) the mean level of extraversion help to interpret this result. In line with our theorizing, Table 3 shows a positive effect of failure feedback when extraversion is high (*b*_high_ = 3.081; *p* = 0.052) and a nonsignificant negative effect of failure feedback when extraversion is low (*b*_low_ = −1.795; *p* = 0.283). Figure 3 illustrates these results.

As shown in Table 2, Model 4, multiple moderation analyses confirm the results described before. In line with our hypotheses, we find a positive interaction between failure feedback and conscientiousness (*β* = 2.165, SE = 1.235, 95% CI = [−0.345, 4.680], *t* = 1.75, *p* = 0.089) and a positive interaction between failure feedback and extraversion (*β* = 1.920, SE = 1.100, 95% CI = [−0.317, 4.156], *t* = 1.74, *p* = 0.090).

## Discussion

The present study set out to expand our knowledge of the differential consequences of failure feedback. Specifically, we examined how conscientiousness and extraversion affect whether individuals learn from failure feedback for subsequent task performance.

Our study reveals no uniform direct effect of failure feedback. This finding is in line with prior research suggesting that failure feedback can motivate individuals to learn and improve but also impair or have no effect on subsequence performance (Kluger and DeNisi, 1996; Steelman and Rutkowski, 2004).

With respect to our hypotheses, we find that when confronted with failure feedback, the high-conscientious participants performed better in a subsequent task than their low-conscientious counterparts. In line with our theoretical reasoning, this result suggests that conscientiousness alleviates the demotivational and discouraging effects of failure feedback. Specifically, this finding reinforces the idea that the achievement and performance orientation associated with conscientiousness (McCrae and Costa, 1987; Barrick and Mount, 1993; Rothmann and Coetzer, 2003) can help individuals overcome the adverse motivational consequences of failure feedback and devote the effort necessary to learn and improve subsequent performance.

Similarly, we also observe that the performance of our study participants confronted with failure feedback benefits from extraversion. As such, our study supports the idea that by providing individuals with self-confidence, action orientation, and the motivation to demonstrate competence (Barrick and Mount, 1991; Bono and Judge, 2004), extraversion facilitates learning from failure feedback.

Overall, our study suggests that conscientiousness and extraversion both serve as buffers against the potentially detrimental effects of failure experiences that are a natural part of the learning process (Ilgen and Davis, 2000; Cianci et al., 2010). This finding resonates with the idea that albeit for different reasons, both personality traits relate to the motivation to learn and adapt (Wang and Erdheim, 2007). With the insights generated, our study complements previous research highlighting how contextual factors, such as the credibility and quality of the feedback provided (Steelman and Rutkowski, 2004), can shape recipients’ reaction to failure feedback. Specifically, the present study contributes to expanding our knowledge of how the characteristics of feedback recipients, such as their regulatory focus (Van-Dijk and Kluger, 2004), influence the consequences of failure feedback.

Given that feedback is an integral part of the effective functioning of organizations (Johnson and Connelly, 2014) and that failure is commonplace in organizational contexts (Morgenroth and Schaller, 2010; Dahlin et al., 2018), our study findings also have practical implications. Previous research indicates that despite their developmental value, supervisors often struggle when providing failure feedback to guide the future efforts of their employees (Steelman and Rutkowski, 2004). Our study suggests that whether supervisors are well advised to proceed with caution when delivering failure feedback hinges on the personalities of their subordinates. Delivering feedback indicating that one’s performance does not meet expectations is unlikely to be an issue when the recipients are either highly conscientious and/or extraverted. When dealing with recipients who score low on conscientiousness and extraversion, in contrast, supervisors may be well advised to pay particular attention to how they deliver failure feedback. Specifically, they may want to build on earlier research (Steelman and Rutkowski, 2004) and deliver such feedback in a particularly considerate and meaningful manner to help alleviate negative reactions.

## Limitations and future research

We acknowledge several limitations related to our study. Due to the COVID-19 pandemic, we conducted our experiment online. Conducting the experiment online allowed us to automate our experiential procedure and increase its uniformity across participants (Dandurand et al., 2008). However, the online setting prevented us from effectively controlling the environment (noise, lighting, technical equipment) in which the individuals participated in the experiment, which may have compromised the internal validity of our study.

While our experimental setting allowed us to manipulate the feedback that participants received, factors that would likely be present in a field setting, such as the opportunity to seek additional information from the feedback provider, were not included in our study. Moreover, we focused on performance in a rather specific, cognitive task. Future research should try to replicate and extend our study findings in a field setting with tasks of various complexity and requiring various types of effort.

Compared to the time horizon for learning and performance in typical work settings, we observed the consequences of failure feedback over a relatively short time frame. Future research should replicate our findings by offering a longer period for learning and improvement and addressing the potentially various short-and longer-term consequences of failure feedback. Our study did not account for the mediators that convey the observed interactive effects of failure feedback and personality. To further expand our knowledge on the consequences of failure feedback, future research should thus elaborate on the processes and emergent states (Mathieu et al., 2008), which can help explain the relationships observed in the present study.

## Data availability statement

The raw data supporting the conclusions of this article will be made available by the authors, without undue reservation.

## Ethics statement

Ethical review and approval was not required for the study on human participants in accordance with the local legislation and institutional requirements. The patients/participants provided their written informed consent to participate in this study.

## Author contributions

TS contributed to conception and design of the study. KT organized the database performed the statistical analysis. KT and TS wrote sections of the manuscript. All authors contributed to manuscript revision, read, and approved the submitted version.

## Conflict of interest

The authors declare that the research was conducted in the absence of any commercial or financial relationships that could be construed as a potential conflict of interest.

## Publisher’s note

All claims expressed in this article are solely those of the authors and do not necessarily represent those of their affiliated organizations, or those of the publisher, the editors and the reviewers. Any product that may be evaluated in this article, or claim that may be made by its manufacturer, is not guaranteed or endorsed by the publisher.
